# A systematic review of long-term cardiotoxic effects of treatment in survivors of childhood acute lymphoblastic leukemia

**DOI:** 10.1186/s40348-025-00196-y

**Published:** 2025-07-04

**Authors:** Paige Johnson, Ellie Whitney, Coleton Evans, Donald Beam

**Affiliations:** 1 Anne Burnett Marion School of Medicine at TCU, Fort Worth, TX USA; 2 Cook Childrens, Fort Worth, TX USA

## Abstract

As progress in medical interventions for childhood cancer advances, the number of survivors of childhood acute lymphoblastic leukemia (ALL) is rising. Presently, the overall survival rate exceeds 90% over a five-year period. With this upward trend in survival rates, there’s a pressing necessity to investigate the enduring impacts of childhood cancer treatment. This systematic literature review focuses on the various long term cardiotoxic effects as a result of these treatments. The authors conducted a systematic review to identify studies that evaluated long-term cardiotoxic effects after anthracycline treatments among survivors of childhood ALL via PubMed search engine. Studies were included if ALL survivors were < 21 years old at the time of ALL diagnosis, an average of > 5 years post diagnosis and/or > 120 weeks post completion of consolidation therapy, compared with a healthy control population, and in remission during the assessment. Compared to matched control populations, survivors of childhood ALL had significantly higher rates of diastolic dysfunction and congestive heart failure. Additionally, female sex, younger age at diagnosis, and higher total dosing of anthracyclines administered during treatment led to significantly higher measures of cardiotoxicity. This study underscores the long-term cardiotoxic effects in ALL survivors, primarily linked to anthracycline use. Inconsistent detection of diastolic dysfunction via conventional echocardiogram necessitates regular monitoring of subclinical markers to prevent premature cardiac aging and heart failure. Future research should prioritize longitudinal studies to assess cardiotoxic effects throughout adult ALL survivors’ lifetimes, aiming to establish comprehensive follow-up guidelines.

## Key points

1. As the number of survivors of childhood ALL increases, there is an equal increase in the number of patients that live long enough to develop negative effects of the treatment regime.

2. Treatment with anthracyclines can lead to early cardiomyopathy and heart failure in long term survivors of childhood ALL.

3. Consistent monitoring of diastolic function and subclinical markers of heart failure can help to diagnose and treat patients preventatively.

## Introduction

As medical advancements in treatments for childhood cancer continue to be made, the overall survival rates of childhood acute lymphoblastic leukemia (ALL) are increasing. Currently, the 5 year overall survival rate is greater than 90%. With these increasing rates, the need for study of the long term effects of childhood cancer treatment is of utmost importance [1]. Childhood acute lymphoblastic leukemia and subsequent treatment has been reported to have a wide range of long term side effects such as cardiotoxicity, metabolic syndrome, obesity, infertility, peripheral neuropathy, subsequent neoplasms, and neurotoxicity [2]. This review will be focusing specifically on the cardiotoxic effects of treatment in long term survivors of childhood acute lymphoblastic leukemia. These effects are predominantly due to the use of anthracyclines as a first line treatment method for childhood ALL [3]. Treatment with anthracyclines is associated with the long term effect of developing congestive heart failure and cardiovascular disease in any patient, regardless of age, treated with this category of drugs. Notably, the later onset of cardiotoxic outcomes commonly occurs in survivors of childhood cancer. The degree of cardiotoxicity of anthracyclines depends on dosing and can have highly varied effects depending on the individual and the dose they received [4]. 

Various parameters can be used to measure cardiotoxicity in patients after anthracycline.

use. Echocardiography and associated measures of left ventricular ejection fraction are mostly used to determine the presence of cardiotoxicity in patients treated with anthracyclines. In addition, measures of myocardial deformation (strain), strain rate, and serum levels of troponin I and T have been shown to be more sensitive measures of detecting early diastolic dysfunction [5, 6]. Early detection is key for providing treatment to potentially reverse the cardiotoxic effects with administration of enalapril and carvedilol [6]. Additionally, some drugs have been shown to be cardioprotective when administered simultaneously with anthracyclines. Specifically, Dexrazoxane is an iron-chelating agent that has been associated with fewer congestive heart failure events and improved left ventricular function. Dexrazoxane’s protective effects work by several mechanisms, one of which is competing with adenosine triphosphate to bind to myocardial topoisomerase beta 2. This prevents the cardiotoxic anthracycline-myocardial topoisomerase 2 beta complexes from forming [7]. 

Given the increased survival rate due to increased treatment with anthracyclines, there is a distinct need for more concise data on the potential long term dangers to survivors of pediatric ALL. This systematic review will evaluate recent studies that measure the cardiac function in long term survivors of acute lymphoblastic leukemia treated with anthracyclines.

## Materials and methods

### Search strategy

The search and evaluation of articles in this systematic review were conducted in accordance with the guidelines set forth by the Preferred Reporting Items of Systematic Reviews and Meta-Analysis statement [8]. The investigators collected articles written in English in a PubMed search from February 4, 2024 to March 18, 2024. To fine-tune the search, disease and outcome specific terms were identified in the MeSH term list and used in the following search algorithm: ((acute lymphocytic leukemia) OR (acute lymphoblastic leukemia) OR (childhood ALL) OR (ALL) OR (pediatric leukemia) OR (leukemia) AND ((cardiovascular abnormalities) OR (cardiologists) OR (heart) OR (heart failure) OR (stroke) OR (cardiotoxic factor) OR (cardiomyopathies) OR (antineoplastic agents/adverse effects). The range of years for studies was limited to 2008–2024. Papers were assessed and eliminated based on title and then abstract review. The remaining articles underwent a full text review by each of the first three listed authors. Each article was then carefully chosen or excluded based on the predetermined selection criteria.

### Selection criteria

Studies included in this systematic review evaluated cardiotoxic outcomes in ALL survivors who met the following eligibility criteria: (1) < 21 years old at the time of ALL diagnosis, (2) an average of > 5 years post diagnosis and/or > 120 weeks post completion of consolidation therapy, (3) compared with a healthy control population, (4) in remission during the assessment. The studies included were either prospective or retrospective. The criteria for a control was deemed necessary to minimize bias, increase reliability, improve validity, and isolate the effects of the desired variable in the study. In retrospective studies, a general population cohort was identified as a control and in prospective studies, a control population needed to have been recruited. The stronger control groups were characterized by primary healthy siblings, but also by healthy subjects that were matched for age, sex, body weight, and body surface area. At least 5 years post diagnosis was determined to be necessary because of the delayed effects of anthracycline treatment and the late development of cardiotoxicity in pediatric populations that were diagnosed at an early age.

### Data extraction

Data extraction was performed collectively by the first three authors and reviewed by the fourth author. The data that was extracted included the country where the study took place, number of controls, number of ALL survivors, mean age at diagnosis, years since treatment, mean age at date of study, and treatment protocol or regimen. Data on five determined outcomes of interest and trends in data were extracted when available: sex, age at diagnosis, diastolic function, heart failure (CHF), and dosing of anthracyclines. It is important to note that these factors are not all absolutely independent from each other, as a decrease in diastolic function can lead to heart failure.

## Results

**Fig. 1:**
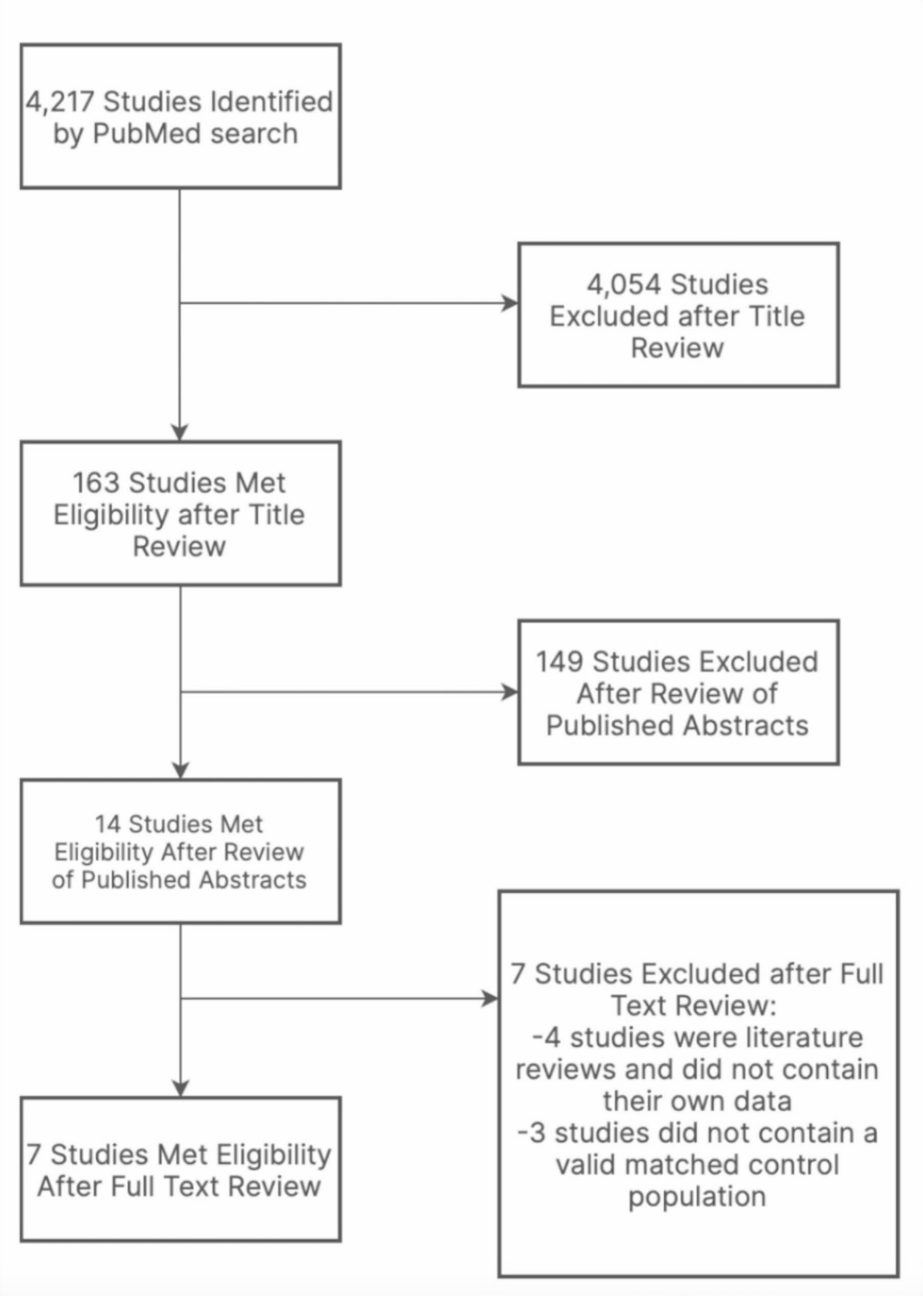
Study Flow Diagram

In total, 4,217 titles and abstracts resulted from the PubMed search. Based on information in titles, 163 studies were identified as pertaining to survivors of ALL, cardiotoxic outcomes, and long term follow up of childhood ALL patients. Abstracts of these 163 papers were each reviewed by the first three authors to determine whether their content met the inclusion criteria. Of the 14 abstracts identified as meeting the inclusion criteria, four literature reviews were eliminated as secondary sources and three studies did not contain a valid matched control population (Fig. 1). Seven studies remained that met the identified inclusion criteria (Table 1).

**Table 1:** Characteristics of included studies

Study Citation Number	Country	Size of Control Population	Size of ALL Survivor Population	Mean Age at Diagnosis	Mean # of Years Since Treatment Cessation	Mean Age at Date of Study
**15**	**Canada**	**8500**	**1479**	**NR**	**10.4**	**NR**
**9**	**Italy**	**50**	**62**	**5.1**	**12.6**	**17.7**
**10**	**Spain**	**58**	**90**	**4**	NR*	**24.6**
**11**	**China**	**66**	**94**	**12.9**	**15**	**22.6**
**12**	**Norway**	**138**	**138**	**5.3**	**23.4**	**28.6**
**13**	**Belgium**	**31**	**37**	**4.8**	**13.3**	**19.8**
**14**	**China**	**42**	**88**	**8**	**15.3**	**24.1**

*Mean number of years since treatment cessation was not reported (NR), but the average number of years since diagnosis of ALL was 18 years, which would exceed the inclusion criteria of at least 5 years since treatment cessation

Across these seven studies, 2,209 patients were survivors of childhood ALL and 8,885 served as healthy matched controls. Six of the seven studies were cross sectional studies, while one paper was a retrospective study using a population-based database of hospitals whose patients were identified using ICD-10 codes [9–15]. The studies had similar inclusion criteria for participation, and reported excluding patients with pre-existing heart conditions, congestive heart failure, and/or congenital heart abnormalities [9–14]. One paper used these preexisting criteria to stratify patients in their results, and therefore only required a minimum age and a diagnosis of ALL for inclusion [12]. 

It is estimated that children with Down Syndrome (DS) have a 10 to 20-fold increased risk of developing acute lymphoblastic leukemia compared to children without DS [16]. To account for this, some studies specified that they excluded patients with DS while one study included 88 children (4% of the studied ALL patient population) with DS [13–15]. In most studies it is not mentioned whether or not these patients were included, but they do not mention DS as a criteria for exclusion [9–12]. 

Per the inclusion criteria for this review, all seven studies included a healthy control population. One study used an exclusively sibling control population while one used controls matched for age & geography in order to match socioeconomic status [10, 15]. One study matched only for sex and age, while two used a mix of sibling and healthy volunteers [9, 11, 14]. The two most thorough matches of control subjects had their control population matched for sex, age, body weight, body surface area, systolic blood pressure, and BMI [12, 13]. Six of the seven papers do not show any significant differences between their controls and the ALL patient population; however, Fernández-Avilés et al. note that there are more females than males in their sibling-only control group. While the sibling control group accounts for immeasurable genetic and environmental factors, the imbalance in sex does bring up potential bias. Regardless of this, the authors state that inverse probability weighting resulted in a good balance of sex and the other relevant covariates [10]. 

All of the studies included ALL survivors who were treated with anthracyclines, but some studies included patients who received both anthracyclines and radiation therapy as is common in treating ALL (Table 2). Several studies included patients who also received Hematopoietic Stem Cell Transplantation in addition to anthracycline treatment and radiation therapy, and these are indicated below.

**Table 2:** Proportion of ALL patient population who received radiation therapy in addition to anthracyclines

Study Citation number	% of ALL Cohort who also received Radiation Therapy	Location of Radiation Therapy	% of ALL cohort who also received Hematopoietic Stem Cell Transplant (HSCT)
**15**	34.6%	Total body including heart field	7.6%
**9**	0%	NA	NA
**10**	3.3%	Thoracic	HCST recipients excluded
**11**	12%	Total body including heart field	HCST recipients excluded
**12**	15%	Cranial or Craniospinal	HCST not mentioned
**13**	24.3%	Cranial	HCST not mentioned
**14**	13%**	Cardiac	12%

**88 of the 100 patients studied were ALL patients, but they included 12 patients treated for Acute Myeloid Leukemia (AML); while most results are stratified, the table showing how many patients were given radiation reflects the portion of total patients included in the study

### Risk of Bias within studies

Despite meeting inclusion criteria for this study, potential biases in the individual papers reviewed must be acknowledged and addressed. Bias was discussed by both the authors of individual papers included in this review as well as the authors of this review. Chellapandian et al. had a very large data set in their retrospective study, but included in Table 1 of their paper that only 1479 of their 1700 person data set for childhood ALL patients were still alive at the time of study. While they excluded patients with preexisting CHF prior to the diagnosis of leukemia and those who died within 30 days from the date of leukemia diagnosis, they still included data of patients who likely had additional diagnoses that led to their death and could have confounded the data set [15]. Fernandez-Aviles et al. address some of their biases in their discussion, where they explain potential implications of having a higher proportion of women in their control group of siblings. Additionally, it was noted by the authors of this review that the group of ALL survivors had a significantly higher proportion of smokers, which could have confounded the results significantly and introduced bias [10]. While Li et al. discuss the many factors that they used to match in their control populations, they did note that their cohort of ALL survivors was significantly shorter than their matched control group. While this may still have implications on the effects of anthracyclines, it is also worth noting that despite the height difference, survivors and controls were matched for weight, body mass index, and body surface area, which may eliminate potential bias [11]. Finally, Cheung et al. explain that while the age and sexes were similar between survivors and controls, the survivors had significantly lower body weight, height, and body surface area. This introduces a significant amount of bias between their 100 survivors and 42 controls, and therefore must be taken into consideration when reviewing their results [14]. Both Chellapandian et al. and Fernandez-Aviles et al. convert the individual doses of various types of anthracyclines into an isotoxic cumulative dose of doxorubicin. However, each study used different coefficients to convert from mitoxantrone, daunorubicin, and epirubicin to doxorubicin [10, 15]. It is difficult to assess the amount of bias created in this review by the discrepancy in this calculation without the full data and calculation information. However, it would be impossible to create such a large sample size without converting from various types of anthracyclines to a uniform cumulative dose. It is also important to note that while it was the intent of this review to analyze the risk of anthracyclines with as few confounding variables as possible, different papers utilized different inclusion criteria and treatments such as hematopoietic stem cell transplantation (HSCT) and different locations of radiation therapy that have undetermined effects on these conclusions. While most studies excluded patients who also received HSCT, Chellapandian et al. indicated that 34.6% of their ALL cohort received radiation therapy, but 7.6% of their cohort also received a HSCT [15]. Similarly, Cheung et al.’s population included anthracycline use in 13% of patients, but 12% of their patients also received a HSCT [14]. 

### Dosing of anthracyclines

All of the papers included in this review evaluated patients who had been treated with various types and various doses of anthracycline chemotherapy. Chellapandian et al. compared equivalent cumulative doses of anthracycline that each patient received. Patients were then stratified as receiving > 250 mg/m2 or < 250 mg/m^2^ [15]. Amigoni et al. found an average cumulative dose across study participants of 228.2 ± 42.3mg/m^2^ and did not compare groups with higher or lower doses [9]. Fernandez-Aviles et al. had an average dose of 138 mg/m^2^ with a range of 72–192 mg/m^2^ [10]. Li et al. had an average cumulative dose of anthracyclines of 227 ± 100 mg/m^2^ [11]. 95% of the patients in Christiansen et al.’s study were treated with anthracyclines. 64 patients were categorized as low dose with a mean isotoxic doxorubicin dose of 120 mg/m^2^. 43 patients were categorized as moderate-high dose with a mean isotoxic doxorubicin dose of 240 mg/m^2^.^12^ Vandecruys et al. explored patients who specifically received low doses of anthracyclines, which they categorized as a mean cumulative dose of < 250 mg/m².^13^ Both Christiansen et al. and Chellapandian et al. divided their patients into two groups. 18 patients received a cumulative dose of 240 mg/m^2^ and 19 received a cumulative dose of 180 mg/m.^2^ [12, 15], Cheung et al. had a mean cumulative anthracycline dose of 218 ± 98 mg/m^2^. The patients were divided into 2 groups. The 19 patients in group I had elevated high sensitivity assay of circulating cardiac troponin T (hs-cTnT) and had a cumulative anthracycline dose of 288 ± 126 mg/m². 81 patients in group II had normal hs-cTnT and a cumulative anthracycline dose of 201 ± 83 mg/m² [14] (Table 3).

**Table 3:** Cumulative dosing of anthracyclines

Study Citation Number	Final Average Anthracycline Dose
15*	> 250 mg/m² OR < 250 mg/m²
**9**	228.2 +/- 42.3 mg/m²
10*	138 mg/m²
**11**	227 +/- 100 mg/m²
**12**	120 mg/m² OR 240 mg/m²
**13**	< 250 mg/m²
**14**	218 +/ 98 mg/m²

*Cumulative doses of various anthracyclines were converted to isotoxic equivalents of doxorubicin using the methods described above

Notably, across the studies reviewed, a higher dose of anthracyclines was associated with increased cardiac damage. Specifically, Chellapandian et al. demonstrated that the higher dose was a significant factor in the development of congestive heart failure [15]. Amigoni et al. explained that patients treated with anthracyclines compared to control had decreased left ventricle dimensions and thickness [9]. Christiansen et al. demonstrated that patients treated with a low or medium/high dose of anthracyclines had decreased systolic function and decreased peak early diastolic velocity of mitral annulus. The group receiving the medium-high doses of anthracyclines showed decreased levels of left ventricle systolic function, diastolic function, and decreased peak oxygen uptake. They also found that VO_2_ inversely varied with dose of anthracyclines [12]. Vandecruys et al. described that decreased dosage (180–240 mg/m^2^) was associated with no cardiac damage after 13.3 years [13]. Cheung et al. found a significant positive correlation between dose and anthracyclines and serum hs-cnTnT in both univariate and multivariate analysis [14]. 

### Diastolic function

Diastolic function is extensively described as an early and particularly sensitive marker for cardiac dysfunction [17, 18]. Diastolic function is measured in various different ways across the articles utilized in this systematic review. Amigoni et al. explained how diastolic function was assessed via transmitral pulsed wave Doppler study, measurement of the ratio between the velocities of E- and A- waves, and the deceleration time of the E-wave in their study population. Diastolic function data in patients with ALL showed reduced left ventricular mass index and lower percent ejection fraction, but there were no significant differences in E/A ratio between the ALL survivor group and the control group [9]. Fernandez-Aviles et al. described how left atrial strain was proposed as a sensitive marker of diastolic dysfunction. Left ventricular diastolic function was evaluated following the American Society of Echocardiography/European Association of Cardiovascular Imaging (ASE/EACVI) algorithm. Left atrial strain was assessed and represented by peak atrial longitudinal strain (PALS), peak atrial contraction strain (PACS), and left atrial strain during the conduit phase (LACS). These culminate in an assessment of the reservoir, contractile, and conduit function of the left atrium. Diastolic function parameters based on variables used in the ASE/EACVI algorithm were within normal limits and there were no significant differences between groups. The findings of left atrial strain demonstrated that PALS and LACS were within the normal limits but were significantly lower in survivors than in controls [10]. Li et al. measured diastolic function by echocardiography examination and M-mode recording of posterior left ventricular wall thickness. Diastolic wall strain was then calculated using the formula DWS = (PWs - PWd)/PWs, where PWs represents posterior wall thickness at end-systole and PWd is posterior wall thickness at end-diastole. Similarly to Amigoni et al.’s findings, the E/A ratio was also calculated to estimate left ventricular filling pressure [10, 11]. Li et al. also measured diastolic myocardial deformation using speckle tracking echocardiography. Results showed that compared with controls, patients had significantly lowered diastolic wall strain, left ventricular global longitudinal, radial, and circumferential early diastolic strain rates, and circumferential late diastolic strain rate [11]. Christiansen et al. measured diastolic function by assessing peak early and late diastolic velocities, the deceleration time of the early wave, E/A ratio, isovolumic relaxation time (IVRT), and left atrial area via pulses Doppler signals of the mitral valve. The results demonstrated that there was diastolic dysfunction in 15% of the ALL survivors [12]. Vandecruys et al. used a Conventional Doppler tracing of the mitral and tricuspid valve to measure peak early (E) and peak atrial (A) velocities and then calculating the E/A ratio. Results of this study showed that in males, the mitral A-wave velocity was significantly associated with a low E/A ratio. This same relationship was not found in the female patient group [13]. Cheung et al. utilized an echocardiographic assessment to measure left ventricular end-systolic and end-diastolic dimensions, and thickness of the interventricular septum and posterior left ventricular wall. Similarly to many of the other papers included, parameters such as early (E) and late (A) diastolic myocardial tissue velocities, E/A ratio, and left ventricular free wall myocardial acceleration during isovolumic contraction (IVA) were also measured. Using speckle tracking echocardiography, global left ventricular longitudinal, radial, and circumferential strain and strain rate were also obtained. Results of this study showed that survivors of ALL had statistically significantly lower left ventricular shortening fraction and ejection fraction when compared to controls, but similar systolic and diastolic function. Among other results, speckle tracking parameters showed a significantly lower diastolic strain rate than controls [14]. Results summarized in Table 4.

### Heart failure

In pediatric populations exposed to anthracycline therapy early in their life, congestive heart failure negatively impacts patient quality of life and has the potential to progress and become fatal [19, 20]. Chellapandian et al. aimed to describe the cumulative incidence and associated risk factors of CHF in children with ALL [15]. Data was obtained via the Pediatric Oncology Group of Ontario Networked Information System (POGONIS). A CHF event was defined as either a single documented hospital admission solely for CHF or one outpatient claim for CHF, followed by at least one additional outpatient claim for CHF within a one-year period. The results of the study showed the cumulative incidence of CHF at various time-intervals post treatment: 0.4% after 6 months, 0.9% after 3 years, 1.2% after 5 years, 1.7% after 10 years, and 2.4% after 15 years. In the other studies reviewed, heart failure was recognized as a potential risk of anthracycline treatment, but was not directly measured; rather, systolic and diastolic function were the measured parameters [9–15]. Results summarized in Table 4.

**Table 4:** Diastolic function and CHF results

Study Citation number	Diastolic Function Measure	Diastolic Function Findings	CHF Measured	Other Cardiac Parameters Measured	Key Findings
**15**	Not measured	Focus on CHF incidence and risk factors.	Yes	CHF Incidence	CHF Incidence was 0.4% after 6 months, 0.9% after 3 years, 1.2% after 5 years, 1.7% after 10 years, and 2.5% after 15 years.
**9**	E/A ratio	No significant differences between the groups.	No	LVEF, LVMi, LV dimensions, PW thickness	Lower EF in survivors. Decreased LV dimensions and thickness in survivors vs. control. LVMi higher with greater age at diagnosis. LVMi and PW thickness significantly related to female sex.
**10**	Left Atrial Strain (PALS, PACS, LACS)	PALS and LACS are significantly lower in survivors than in controls.	No	Left atrial volume, E/A ratio	Conventional diastolic function parameters were within normal limits, no significant differences between groups.
**11**	DWS (via PW thickness), DSR, E/A ratio	Survivors had significantly lowered DWS, LV global longitudinal, radial, circumferential early DSR and circumferential late DSR vs. controls. No significant differences in the E/A ratio between the groups.	No	Calibrated integrated backscatter (myocardial fibrosis).	DWS did not correlate with age, time since chemo, or anthracycline dose. Average calibrated integrated backscatter correlated negatively with age at study.
**12**	Peak E and A velocities, E/A ratio	Diastolic dysfunction in 15% of ALL survivors. Decreased peak E wave velocity in survivors vs. controls. No significant difference in the E/A ratio between groups.	No	LVEF, LVFS, GS, peak oxygen uptake (VO_2_)	Significantly decreased systolic function parameters (LVEF, LVFS, GS) in survivors. Inverse correlation between VO_2_ and anthracycline exposure.
**13**	Peak E and A velocities, E/A ratio	No clinically relevant late cardiotoxicity reported. Significant difference in the E/A ratio observed in male survivors but not female survivors.	No	Left Ventricular Dimensions, LVEF, IVRT	No significant difference in left ventricular dimensions in either gender. Significantly lower and higher LVEF and IVRT, respectively, observed in male population but not female population.
**14**	E/A ratio, DSR	Significantly lower DSR in survivors vs. controls. No significant differences in the E/A ratio between the groups.	No	LVFS and LVEF, hs-cTnT	Survivors had statistically significant lower LVFS and LVEF vs. controls. Elevated hs-cTnT associated with worse LV systolic and diastolic myocardial deformation.

Abbreviations: LV, Left Ventricular; LVEF, Left Ventricular Ejection Fraction; LVMi, Left Ventricular Mass Index; PW, Posterior Wall; PALS, Peak Atrial Longitudinal Strain; PACS, Peak Atrial Contraction Strain; LACS, Left Atrial Strain during the conduit phase; DWS, Diastolic Wall Strain; DSR, Diastolic Strain Rate; LVFS, Left Ventricular Fractional Shortening; GS, Global Strain; IVRT, Isovolumetric Relaxation Time

### Age

While all studies in this review identified the average age at diagnosis for their respective ALL survivor cohort, a few classified age at diagnosis to be a risk factor for developing cardiotoxicity [10, 12, 15]. Chellapandian et al. demonstrate that age < 1 year at cancer diagnosis was a significant independent risk factor for congestive heart failure in the ALL cohort [15]. In patients given low anthracycline doses, Amigoni et al. found that the left ventricular mass index had a direct and significant relationship to the patients’ age at the time of ALL diagnosis: it tended to be higher for greater age at diagnosis and vice versa [9]. According to Li et al., diastolic wall strain, a measure of myocardial stiffness, did not correlate with age, age at diagnosis, time since completion of chemotherapy, or mean cumulative anthracycline dose. However, the average calibrated integrated backscatter, a measure of myocardial fibrosis, correlated negatively with age at study, but not with age at diagnosis, time since completion of chemotherapy, and mean cumulative anthracycline dose [11]. Alternatively, some studies found no direct correlations at all with age and adverse cardiac outcomes [12, 13]. Christiansen et al. did not report a direct correlation with age, but does state that although the changes are small, the survivors in their study are still young and impaired LV diastolic function may be a sign of premature aging that can lead to clinical impairment and require medical interventions later in life [12]. Vandecruys et al. explained that when looking among patients who received a low dose of anthracyclines, they did not find a young age at treatment, especially younger than 4 years, to be a risk factor for developing cardiotoxicity [13]. 

### Biological sex

Biological sex was stratified and tested as a potential risk factor for cardiotoxicity in three of the studies included in this review. Most studies agree that female sex is a risk factor for developing anthracycline related cardiotoxicity later in life, but certain low doses of anthracycline have differing results. When determining risk factors for congestive heart failure, Chellapandian et al. found that in univariate analyses, female sex was significantly associated with congestive heart failure [15]. Similarly, in the stepwise analysis on the whole population studied by Amigoni et al., both left ventricular mass and posterior wall thickness persisted to be significantly related with biological sex. This population received what is considered a low dose of anthracyclines (cumulative dose 228.2 ± 42.3 mg/m2), but still see that females are at greater risk of cardiotoxic effects from these doses [9]. However, in a similar study of the long term outcomes of low dose anthracycline use (cumulative dose 180 mg/m² or 240 mg/m²), Vandecruys et al. did not find female sex to be a risk factor and they attribute this to the fact that the previously found correlation in an older study is weaker in the low cumulative anthracycline dose range [13, 21]. 

## Discussion

In this systematic review, the long term cardiotoxic effects of treatment in survivors of childhood ALL were explored. Anthracycline dose proved to be a key risk factor for cardiotoxic effects as exemplified through various methods of measuring cardiac function. Most of the papers included in this review evaluated the post-treatment cardiac function through measurement and evaluation of diastolic dysfunction in the heart. Additionally, the impacts of age and sex as potential risk factors for increased likelihood of late cardiotoxicity and congestive heart failure were discussed.

Anthracyclines have been proven to cause cardiotoxic effects both in the short and long term when used as a chemotherapeutic agent [4]. The results of this review indicated that anthracyclines cause a dose dependent cardiotoxic effect [9, 10], [12–15]. Specifically, patients receiving higher doses of anthracyclines had higher serum levels of hs-cnTnT indicating cardiac damage [14]. Additionally, anthracycline use was associated with decreased left ventricular dimensions and thickness [22]. The mechanism by which anthracyclines cause cardiotoxicity has been studied and is likely due to the induction of lipid peroxidation in all tissues as well as oxidative stress due to anthracycline-induced free radical generation and activation of nitric oxide synthase [22, 23]. Ultimately, these effects lead to DNA damage and subsequent myocyte death resulting in irreversible myocardial damage [4]. Additionally, the dose dependent effects of cardiotoxicity found by Christiansen et al. are corroborated by Arola et al. [12, 24]. A larger dose of anthracycline would lead to the production of more free radicals, increased oxidative stress to the cardiac myocytes and ultimately greater damage [25]. While there is not a safe dose level to guarantee prevention of cardiac myocyte damage, a level of < 250 mg/m2 has been shown to have lower incidence of myocyte damage and cardiotoxicity, as described by Chellapandian et al. and Vandecruys et al. [13, 15, 24, 26] In both studies, patients receiving lower doses of anthracyclines were found to have no symptoms of congestive heart failure or clinically relevant cardiac damage, respectively [13, 15]. However, Christiansen et al. did find some markers of decreased systolic and diastolic function in the low dose group [12]. As such, it is important for patients who receive any amount of anthracyclines to be aware of these effects and receive appropriate screening and treatment when indicated. When considering these effects, it is important to note that the dosage of anthracyclines in all studies considered is generally higher than the current Children’s Oncology Group recommendation. Their lowest threshold for screening begins at a cumulative dose > 100 mg/meters squared [27].

To mitigate cardiotoxic effects, potential mechanisms for preventing this oxidative stress induced cardiac damage have been explored. The PEGylated liposomal form of doxorubicin has shown to have decreased circulating doxorubicin and uptake is selective for tumor cells. It has reduced cardiac damage even at high doses in some studies [4]. 

There is discourse among researchers whether diastolic function indices are altered in long-term survivors of ALL treated with cumulative dosing of anthracyclines. Diastolic function has been demonstrated to be one of the earliest manifestations of cardiac damage in these patient populations [17, 18]. Of the papers included in this systematic review, three found that diastolic function indices were not consistently altered or were not significantly different between the experimental and control groups [9, 10, 13]. Contrarily, three papers reported data that found significant reduction of parameters of diastolic function [11–14]. Fernandez-Aviles et al. report that conventional echocardiographic diastolic parameters might be insufficient to detect the earliest stages of diastolic dysfunction [28]. This demonstrates the importance of testing for subclinical markers in addition to conventional echocardiogram [10]. This is also identified by Vandecruys et al., who explained that while there were no clinically relevant cardiotoxicity effects found, there were subclinical cardiac abnormalities present in a third of these patients [13]. The lack of signs of early diastolic dysfunction may occur, in part, because the cardiac damage that is induced by anthracyclines consists of a reduction in the cardiac mass. This result is also identified by Amigoni et al. While this may impair cardiac contractility, it has little adverse effects on the ability of the cardiac walls to distend and expand during diastole [9]. There is also a relationship noted, particularly in Fernandez-Aviles et al. and Christiansen et al., between patients exposed to higher doses of anthracyclines and an earlier subclinical stage of diastolic dysfunction [10, 12]. This relationship is particularly important when considering the worsening of diastolic function with aging-related changes in left ventricular filling and function. This is a sign of premature aging resulting in earlier development of CHF [29, 30]. 

Diastolic wall strain was shown to be an important parameter of diastolic function. Li et al. showed a positive correlation between diastolic wall strain and indices of left ventricular diastolic function [11]. Reduced diastolic wall strain is suggestive of increased left ventricular myocardial stiffness. It is probable that there is a shared factor contributing to this pathological alteration, such as myocardial fibrosis, which could potentially modify the intrinsic properties of the myocardium during diastole. The mechanism of action of the stiffness is likely due to excessive accumulation of collagen within the myocardium, increasing elasticity, and compromising relaxation during diastole [11]. Cheung et al. contributed by measuring hs-cTnT levels, a subclinical marker that is useful in the detection of subtle myocardial damage. Increased hs-cTnT levels has been associated with worse left ventricular systolic and diastolic myocardial deformation. The hs-cTnT levels were also shown to be associated with cardiovascular risk factors such as age, blood pressure, renal function, current smoking, and left ventricular hypertrophy. Continuous stress-induced myocardial strain results in persistent damage, causing temporary alterations in cell membrane permeability and the release of cTnT in patients following anthracycline therapy [14]. 

While most studies had some measure of diastolic dysfunction, additional variables were shown to be significant in modifying the degree of cardiotoxic effects in survivors of ALL, including sex and age. Three papers in this review assessed sex as a risk factor [9, 13, 15]. Two agreed that female sex is indeed a risk factor for developing late cardiotoxicity [9, 15]. As is the case with many drugs, anthracycline clearance is reduced with increased body fat; consequently, females, who typically have more body fat may have greater anthracycline exposure compared to males receiving the same dose. This has been demonstrated with doxorubicin (DOX), where it has been shown that males had significantly higher drug clearance when compared to females after adjusting for body surface area [31]. Since DOX does not distribute to fat tissues, relatively higher DOX concentrations can be achieved in other tissues such as the heart [32]. Chellapandian et al. note a similar assumption in their discussion of their study limitations. They discussed how their study lacked body mass index data and measures of adiposity and thus could only hypothesize reasons for the sex effect observed in patients with ALL [15]. 

In addition, females have elevated levels of carbonyl reductase 1, an enzyme involved in converting doxorubicin to its active metabolite, doxorubicinol. This partly explains the enhanced cardiotoxicity in females. Although many clinical studies have attempted to determine the role of sex as a risk factor for anthracycline induced cardiotoxicity, the clinical data is still inconclusive [21]. 

While the results are conflicting, Vandecruys et al. do note in their discussion on sex and cardiotoxicity that their lack of significant results is a finding from a small cohort that needs further study. Compared to the studies with significant findings by sex, their cohort is significantly smaller (See Table 1) [13]. In a larger population of survivors of childhood ALL, it does seem to be a significant finding potentially due to the effects of anthracycline metabolism [15]. 

While ALL is widely understood to be a childhood cancer, some studies in this review suggest that the age of the child is also important when predicting cardiotoxic outcomes, with two studies finding a significant correlation and three finding a lack thereof [9, 11–13, 15]. Generally, acute lymphoblastic leukemia in infants has unique disease biology, and infants receive very different treatment approaches compared to children aged one year [15]. The peculiar clinical and biological characteristics of acute leukemia in infants differ markedly from those of older children. These patients usually present with high initial white blood cell counts, massive organomegaly, early central nervous system involvement, and leukemia cutis [33]. Thus, infant leukemia tends to be more aggressive with poor prognosis. The effect of age persisted after adjustment for anthracycline dose which suggests that other chemotherapy used in infant ALL (including alkylators) may have an important impact on CHF risk [15]. Another aspect according to Amigoni et al. is that the anthracycline related inhibition of myocyte growth at a crucial time for organ development is associated with long term irreversible consequences [9]. While these studies agree that age at diagnosis did play a significant factor, several studies offer a different analysis. These studies do not offer discussion on why their lack of results may be.

## Conclusion

In conclusion, long-term cardiotoxic effects have been demonstrated in survivors of acute lymphoblastic leukemia. The use of anthracyclines is a significant risk factor for the development of cardiotoxicity in a dose dependent manner. Evidence of diastolic dysfunction is often inconsistently obtained via conventional echocardiogram, therefore, subclinical markers should be consistently measured in these patient populations to prevent premature cardiac aging and development of congestive heart failure. Additionally, this exemplifies our need for better imaging modalities. Age at diagnosis and female sex are often, but not unanimously, associated with increased cardiotoxicity, but this mechanism is not fully understood. Areas of future study should include very long term follow up on adult survivors of ALL periodically to assess cardiotoxic effects throughout their lifetime, rather than a cross sectional data point. Despite the advancements in treating ALL and increasing survival rates, the research on the implications of this effective treatment in the future and appropriate guidelines for follow up are still inconclusive and should be investigated further.

## Electronic Supplementary Material

Below is the link to the electronic supplementary material.

## Data Availability

No datasets were generated or analysed during the current study.
